# Photoprotection role of melanin in the human retinal pigment epithelium. Imaging techniques for retinal melanin

**Published:** 2020

**Authors:** Marina Istrate, Brigitha Vlaicu, Marioara Poenaru, Mihai Hasbei-Popa, Mădălina Casiana Salavat, Daniela Adriana Iliescu

**Affiliations:** * “Victor Babeș” University of Medicine and Pharmacy, Timișoara, Romania; ** Department of Hygiene, “Victor Babeș” University of Medicine and Pharmacy, Timișoara, Romania; *** ENT Department, “Victor Babeș” University of Medicine and Pharmacy, Timișoara, Romania; **** “Iuliu Hațieganu” University of Medicine and Pharmacy, Cluj-Napoca, Cluj, Romania; ***** Department of Physiology II, “Carol Davila” University of Medicine and Pharmacy, Bucharest, Romania

**Keywords:** eumelanin, pheomelanin, retinal pigment epithelium, antioxidant properties, imaging techniques

## Abstract

The human eye is made up of multiple layers of pigmented tissues that have in componence melanin. In the eye, one can separate melanosomes from various embryonic origins. The pigment-producing cells in the stroma of the iris, ciliary body and the choroid (uveal melanocytes) are neural crest derivatives. On the other hand, ciliary, iris and retinal pigment epithelial cells are developed from the neural ectoderm. One universally accepted role of melanin is to react as neutral-density filter in scattering light. Melanin acts as a free radical stabilizer and has the ability to absorb near-infrared, visible light and UV radiation.

This paper reviews the current knowledge on ocular melanin, including ocular melanogenesis, roles of melanin in retinal metabolic processes and some imaging techniques that identify melanin in the retina.

## Introduction

**Fig. 1:**
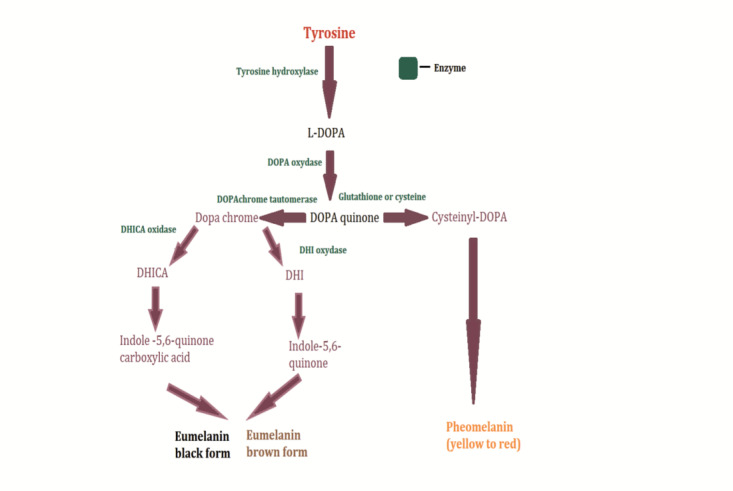
Melanin synthesis pathway

The morphology of the human eyeball can be fractionated into three basic tunics: the fibrous tunic, also known as tunica fibrosa oculi (cornea and sclera), the vascular tunic or the “uvea” (iris, ciliary body and choroid) and the nervous tunic (retina) [**1**,**2**]. Melanin is normally present in the uveal coat and retinal pigment epithelium (RPE). Melanocytes in the ciliary body and iris are identified in the stroma. Choroidal melanocytes are located in choroidal stroma and suprachoroidal space. The choroidal melanocytes function has not yet been fully elucidated [**3**-**6**]. Melanin is also present in the pigment epithelium cells [**1**]. The melanin pigment is synthetized in a specialized cluster of cells also known as melanocytes. Melanin is produced through an intricate chemical way (**Fig. 1**). Its synthesis precursor is an aromatic amino acid: α-tyrosine [**7**]. 

**Melanin and ocular melanogenesis**

The retinal pigment epithelium is naturally intensely pigmented. Melanin in the retinal pigment epithelium is mostly eumelanin. There are two types of eumelanin, which are brown and black, synthetized from levodopa or tyrosine. The melanin amount in the RPE reduces importantly in aged eyes. Consequently, melanin biosynthesis is null or tiny in adult human RPE cells. The turnover of retinal melanosomes is not fully elucidated [**8**-**11**]. In the uveal layer, the features of melanin pigment differ with the iris color. Uveal melanocytes consist both of eumelanin and pheomelanin, but pheomelanin is frequently present more than eumelanin [**12**-**14**]. Pheomelanin is a xanthous pigment that is synthetized when glutathione or cysteine is included in the oxidation phase of levodopa [**7**]. 

**Retinal pigment epithelium**

The retinal pigment epithelium (RPE) is a layer of cuboidal epithelial cells placed between choriocapillaris and the outer segment of the photoreceptor cells. The human eye consists of approximately 4-6 million RPE cells per eye. The RPE cells are polarized cells. They have microvilli on their apical portions that engage with the outer segment of photoreceptor cells. The basal area (which is adjacent to Bruch’s membrane) has many infoldings. In essence, the RPE contributes to the blood-retina barrier. Besides the organelles found in other cells, the RPE has melanin granules and phagosomes [**1**,**2**]. The cytoplasm of the RPE cells is rich in pigment granules (melanosomes). These organelles evolve in situ during the genesis of the optic cup and firstly develop as nonmelanized premelanosomes. Their development differs strongly from that of the pigment granules in uveal melanocytes [**2**]. Melanin in the retinal pigment epithelium is a mixed polymer synthesized from α-tyrosine [**15**]. Melanin pigment has intrinsic, indole semiquinone-like radicals in its componence [**16**,**17**]. Extrinsic indole semiquinone-like radicals are generated under visible and ultraviolet irradiation [**18**]. Retinal melanin has an important photoprotective function by neutralizing reactive oxygen species (ROS) and reducing free radicals damage [**19**,**20**]. Also, the RPE is abundant in peroxisomes, suggesting that it is effective in detoxifying a lot of free radicals. Pigment granules are plentiful in the cytoplasm of pigment epithelium cells, principally in the apical portion. During growth, stimulation of the tyrosinase promoter triggers the initiation of melanogenesis in this cell and evidences the attribution of the neuroectoderm to pass into RPE. However, most melanogenesis process befalls before birth. Melanin synthesis process in the retinal pigment epithelium occurs throughout life at a slow rate. *With advancing age*, the melanin granules interfere with lysosomes. Thus, the posterior pole of an elderly human is less pigmented. Melanin is a free-radical stabilizer. One major capacity of melanin is to behave as a neutral-density filter for scattered light [**2**]. On the other hand, retinal pigment epithelium contains lipofuscin – yellow-brown pigment granules composed of lipid debris of lysosomal digestion. In comparison with melanin, lipofuscin in the RPE enhances after birth and continues to increase with age. Light damage mediated through photosensitized oxidations has been considered to result in lipid peroxides. This suggests that there could possibly be a liaison between melanin concentration and lipofuscin enhancement in the RPE [**21**-**23**]. 

**Major roles of the retinal pigment epithelium**

- Phagocytosis of photoreceptor outer-segment discs,

- Transport of nutrients and ions to photoreceptors, 

- Visual pigment regeneration,

- Decreasing light scatter,

- Blood-retinal barrier,

- Synthesis and maintenance of interphotoreceptor matrix,

- Synthesis and secretion of growth factors,

- Immune modulation [**2**].

**Imaging retinal melanin: current technologies**

Ocular imaging has numerous benefits, both to make better patient carefulness and to realize scientific research. Quantifying melanin in the eyeball ensures data regarding to the global health status of the RPE and of adjacent anatomical parts. The published literature recommends a series of techniques that are used to identify ocular melanin: optical coherence tomography (OCT), near-infrared autofluorescence imaging technique (NIR-AF), photoacoustic imaging, fundus photography and fundus reflectometry. 

**Optical coherence tomography (OCT)**

Optical coherence tomography is a three-dimensional imaging practice that is successfully used in ophthalmology for imaging the human eye. OCT is built on low-coherence interferometry utilizing near-infrared light. OCT subtypes such as polarization-sensitive OCT (PS-OCT) and photothermal OCT (PT-OCT) have been invented to recognize melanin using its polarization and absorption qualities. PS-OCT emerges to be a useful completion of OCT. Depolarization in the retinal pigment epithelium was utilized as a tissue particular contrast. The allotment of the depolarization of the RPE was based to be correspondent with the dispersion of the melanin pigment [**24**]. On the other hand, photothermal OCT identifies optical absorbers in tissues better than OCT. Photothermal OCT is based on the heat-producing properties of antibody-conjugated gold nanoparticles to realize molecular contrast. PT-OCT has the benefit of the photothermal impact, where photons absorbed by the melanin pigment are emanated as heat [**25**]. Swept-source OCT (SS-OCT) is a more recent technique that confers important benefits for analyzing ocular tissues. Backscattered light is the functional principle of SS-OCT. This is compared to a reference beam that, when overlapped, makes an interference pattern. SS-OCT has a better penetrance for retinal melanin and choroid in contrast to other OCT imaging techniques [**26**]. 

**Photoacoustic imaging technique**

This imaging technique uses ultrasounds to underline optical absorbers. Photoacoustic imaging applies a pulsed-laser and an ultrasonic transducer. The light emitted by pulsed laser is absorbed by retinal melanin pigment. The photoacoustic imaging signal severity is correlated with melanin absorption, which makes it possible to differentiate the signal from retinal pigment epithelium and the choroid [**27**].

**Fundus photography**

Fundus photography makes a bidimensional image of the retina. This imaging practice can show changes in pigmentation. However, images are only qualitative and it is not possible to differentiate retinal and choroidal melanin [**28**]. To obtain quantitative information, fundus reflectometry was used. 

**Fundus reflectometry**

The method of fundus reflectometry is practiced with a retinal densitometer. Retinal densitometer includes a light emitter and several filters that can modify the light’s wavelength, which comes into the eye and an analyzer (photomultiplier) for the light exiting the eyeball. This imaging technique ensures quantitative details about melanin pigment distribution in the posterior pole [**29**].

**Near-infrared autofluorescence imaging (NIR-AF)**

Scanning laser ophthalmoscopy (SLO) is an alternative method of visualizing the posterior pole. It uses the mechanism of confocal laser scanning microscopy for screening examinations of cornea and retina. SLO has activated near-infrared autofluorescence imaging mode of the posterior pole (NIR-AF). SLO obtains bidimensional pictures of the eye fundus. A pinhole can gather light from a different layer of the eye fundus. Two endogenous fluorochromes are imaged: lipofuscin and melanin. However, it is tough to evaluate melanin spread using NIR-AF. This technique is not enough to differentiate retinal melanin from choroidal melanin [**30**].

**Protective effects of ocular melanin**

Melanin is an effective absorbent of infrared light, visible light and ultraviolet radiation [**31**]. The melanin pigment can annihilate over 99.9% of the absorbed UV radiation [**11**]. In the anterior pole of the eyeball, the melanocytes block visible and infrared light and ultraviolet radiation. In the posterior pole (RPE), melanosomes decrease the photo oxidative stress and act like a shield against the scattered light [**32**]. Melanin is a free-radical stabilizer and can dismiss numerous toxins [**2**].

## Conclusions

Melanin pigment is normally found in the uveal coat and RPE, and acts as a defender of the photoreceptor cells to keep the retinal health. Melanin granules in the retinal pigment epithelium (RPE) have many important functions, which have not yet been completely elucidated. Melanin protects the cells from oxidative stress injury. This pigment is proficient to stabilize free radicals and decreases cytotoxic lipid peroxidation. Thus, melanin decreases light toxicity and protects against cytotoxic impacts caused by inflammatory processes. Melanin has the ability to absorb infrared light, visible light and ultraviolet radiation.

**Acknowledgments**

No financial disclosure.

**Conflict of interests**

The authors declare no conflict of interest.

## References

[1] Strauss O (2005). The retinal pigment epithelium in visual function. Physiological Reviews.

[2] (2011-2012). Fundamentals and Principles of Ophtalmology. American Academy of Ophthalmology.

[3] Snell RS, Lemp MA (1998). Clinical Anatomy of the Eye.

[4] Bumstead KM, Barnstable CJ (2000). Dorsal retinal pigment epithelium differentiates as neural retina in microphthalmia mouse. Invest. Ophthalmol. Vis. Sci.

[5] Rak DJ, Hardy KM, Jaffe GJ, McKay BS (2006). Ca(++)-switch induction of RPE differentiation. Exp. Eye Res.

[6] Hu DN (2005). Photobiology of ocular melanocytes and melanoma. Photochem. Photobiol.

[7] Slominski A, Tobin DJ, Shibahara S, Wortsman J (2004). Melanin pigmentation in mammalian skin and its hormonal regulation. Physiol. Rev.

[8] Wakamatsu K, Ito S (2002). Advanced chemical methods in melanin determination. Pigment Cell Res.

[9] Clancy CMR, Simon JD (2001). Ultrastructural organization of eumelanin from Sepia officinalis measured by atomic force microscopy. Biochemistry.

[10] Liu Y, Simon JD (2003). Isolation and biophysical studies of natural eumelanins: Applications of imaging technologies and ultrafast spectroscopy. Pigment Cell Res.

[11] Meredith P, Sarna T (2006). The physical and chemical properties of eumelanin. Pigment Cell Res.

[12] Prota G, Hu DN, Vincensi MR, McCormick SA, Napolitano A (1998). Characterization of melanins in human irides and cultured uveal melanocytes from eyes of different colors. Exp. Eye Res.

[13] Berendschot TT, Willemse-Assink JJ, Bastiaans M, de Jong PT, van Norren D (2002). Macular pigment and melanin in age-related maculopathy in a general population. Invest Ophthalmol Vis Sci.

[14] Wakamatsu K, Hu DN, McCormick SA, Ito S (2008). Characterization of melanin in human iridal and choroidal melanocytes from eyes with various colored irides. Pigment Cell Res.

[15] Hong L, Simon JD, Sarna T (2006). Melanin structure and the potential functions of uveal melanosomes. Pigment Cell Res.

[16] Boulton M, Rozanowska M, Rozanowski B (2001). Retinal Photodamage. J Photochem. Photobiol.

[17] Burke JM, Henry MM, Zareba M, Sarna TI (2007). Photobleaching of melanosomes from retinal pigment epithelium: I. Effects on protein oxidation. Photochem. Photobiol.

[18] Schraermeyer U, Heimann K (1999 ). Current understanding on the role of retinal pigment epithelium and its pigmentation. Pigm. Cell Res.

[19] Rozanowska M, Sarna T, Land E, Truscott T (1999). Free radical scavenging properties of melanin interaction of eu- and pheo-melanin models with reducing and oxidising radicals. Free Radical Biol. Med.

[20] Pane AR, Hirst LW (2000). Ultraviolet light exposure as a risk factor for ocular melanoma in Queensland, Australia. Ophthalmic Epidemiol.

[21] Samokhvalov A, Hong  L, Liu Y, Garguilo J, Nemanich  RJ, Edwards GS, Simon  JD (2005). Oxidative potentials of human eumelanosomes and pheomelanosomes. Photochem. Photobiol.

[22] De Leeuw SM, Smit NP, Van Veldhoven M, Pennings  EM, Pavel S, Simons  JW, Schothorst  AA (2001). Melanin content of cultured human melanocytes and UV-induced cytotoxicity. J. Photochem. Photobiol. B. Biol.

[23] Bok D (2005 ). Evidence for an inflammatory process in age-related macular degeneration gains new support. Proc. Natl Acad. Sci. USA.

[24] Pircher M, Hitzenberger CK, Schmidt-Erfurth U (2011). Polarization sensitive optical coherence tomography in the human eye. Prog Retin Eye Res.

[25] Adler DC, Huang SW, Huber R, Fujimoto JG (2008). Photothermal detection of gold nanoparticles using phase-sensitive optical coherence tomography. Opt Express.

[26] Yasuno Y, Hong Y, Makita S (2007 ). In vivo high-contrast imaging of deep posterior eye by 1-micron SS-OCT and scattering OCTA. Opt Express.

[27] Silverman RH, Kong F, Chen Y, Lloyd HO, Kim HH, Cannata JM (2010 ). High-resolution photoacoustic imaging of ocular tissues. Ultrasound Med Biol.

[28] Panwar N, Huang P, Lee J, Keane PA, Chuan TS, Richhariya A (2016 ). Fundus photography in the 21st century—a review of recent technological advances and their implications for worldwide healthcare. Telemed J E Health.

[29] Hammer M, Schweitzer D (2002 ). Quantitative reflection spectroscopy at the human ocular fundus. Phys Med Biol.

[30] Demos SG, Gandour-Edwards R, Ramsamooj R, White R (2004 ). Near-infrared autofluorescence imaging for detection of cancer. J Biomed Opt.

[31] Sarna T, Swartz HA (1998 ). The physical properties of melanin. In The Pigment System: Physiology and Pathophysiology (Edited by Nordland JJ, Boissy RE, Hearing VJ, King RA, Ortonne JP).

[32] Liu Y, Hong L, Wakamatsu K, Ito S, Adhyaru BB, Cheng CY, Bowers CR, Simon JD (2005). Comparisons of the structural and chemical properties of melanosomes isolated from retinal pigment epithelium, iris and choroid of newborn and mature bovine eyes. Photochem. Photobiol.

